# Acute Ischemic Colitis due to Oral Phenylephrine

**DOI:** 10.14309/crj.0000000000000459

**Published:** 2020-09-29

**Authors:** Emran El-Alali, Tarik Alhmoud

**Affiliations:** 1 Department of Medicine, Anne Arundel Medical Center, Annapolis, MD; 2 Promedica Digestive Health Care (affiliated with ProMedica Toledo Hospital, Toledo, OH), Sylvania, OH

## Abstract

Oral phenylephrine is a commonly used over-the-counter nasal decongestant drug. It is usually taken for symptomatic relief (in combination drug products) for upper respiratory tract infections, allergic rhinitis, or sinusitis. Adverse cardiovascular effects of intravenous phenylephrine, including organ ischemia, are well known; however, oral phenylephrine is rarely associated with significant adverse effects. We describe the first case of acute ischemic colitis in a young patient due to over-the-counter oral phenylephrine, which was taken as a nasal decongestant. We reviewed the literature of colonic ischemia related to the use of systemic nasal decongestants phenylephrine and pseudoephedrine.

## INTRODUCTION

Ischemic colitis (IC) is caused by sudden reduction in colonic perfusion leading to ischemic injury. IC is most commonly caused by nonocclusive colonic ischemia and less often due to acute arterial occlusion or mesenteric venous thrombosis.^1^ The prevalence of acute intestinal vascular insufficiency, including IC, is 406 of 100,000.^2^ Various medications can cause IC through vasoconstriction, hypotension, thrombogenic/procoagulant effect, or direct colonic toxicity.^1,3,4^ Over-the-counter (OTC) systemic nasal decongestants include the alpha-adrenergic agonists pseudoephedrine and oral phenylephrine, which are used for symptomatic relief of nasal congestion in common cold, allergic rhinitis, and sinusitis. Alpha-adrenergic agonists work via arterial vasoconstriction, which can potentially lead to tissue ischemia of various organs. Pseudoephedrine is a more potent decongestant compared with phenylephrine and has been linked to IC in several case reports. Intravenous phenylephrine is used to treat shock and hypotension and is known to cause cardiovascular adverse effects and tissue ischemia. Such side effects are rare with oral phenylephrine.

## CASE REPORT

A 34-year-old White man with no significant medical history presented to the emergency department with a 2-day history of lower abdominal pain. Pain was associated with watery diarrhea, which had progressed to hematochezia. The patient had no nausea, vomiting, fever, or chills. He was admitted to the hospital for further management.

The patient used multiple OTC DayQuil LiquiCaps (containing phenylephrine 10 mg per LiquiCaps) over 2 days before symptoms onset to relieve nasal congestion and cold symptoms. He did not use other OTC or prescription medications. The patient reported no family history of hypercoagulation disorders. He chews tobacco occasionally and does not drink alcohol or use illicit drugs.

Vital signs were normal. Body mass index was 43 kg/m^2^. Heart rate and rhythm were normal and regular. Bowel sounds were normal. Abdomen was distended and showed left lower quadrant tenderness, no peritoneal signs, and no hepatosplenomegaly. White blood cell count was elevated (11.9 × 10^9^/L), and hemoglobin was normal (15.0 g/dL). Serum creatinine, electrolytes, and liver enzymes were normal. Enteric pathogen panel was negative. Urine drug screen was negative for illicit drugs. Abdominal and pelvic computed tomography showed mural thickening of the descending colon with infiltrative changes in the mesentery compatible with left-sided colitis.

**Figure 1.**
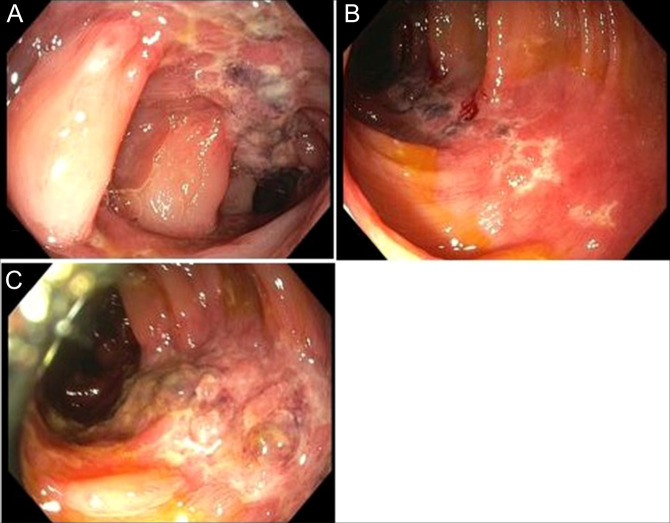
Endoscopy showing edema, erythema, friability, loss of vascularity, erosions, and ulcerations in the sigmoid colon (A and B) and descending colon (C).

During hospital stay, the patient was started on intravenous fluids and he maintained normal blood pressure, heart rate, and hemoglobin level. Gastroenterology consultation was requested, and colonoscopy demonstrated segmental moderate inflammation of the descending and sigmoid colon, characterized by mucosal congestion, erythema, erosions, friability, loss of vascularity, serpentine, and shallow ulcerations suggestive of IC vs, less likely, Crohn's disease (Figure 1).

Histologic examination confirmed the diagnosis of IC. The patient was managed conservatively and was subsequently discharged from the hospital after resolution of symptoms. He was advised to avoid all phenylephrine-containing OTC products and to quit tobacco use. Outpatient mesenteric duplex study revealed no impairment to visceral circulation. He remained asymptomatic and was doing well after 2 months, during an outpatient office visit.

## DISCUSSION

We report a case of IC in a young man after the use of oral phenylephrine that was taken as a decongestant for common cold. IC was likely induced by the vasoconstrictive effect of the alpha-adrenergic receptor agonist phenylephrine, probably aggravated by coexisting smokeless tobacco use. Although the patient had obesity (body mass index of 43 kg/m^2^), he had no hypertension, heart disease, diabetes, or previous thromboembolic events, and his drug screen was negative for illicit drugs that are associated with IC (cocaine or amphetamines). A mesenteric duplex showed no vascular occlusions or impairment to visceral circulation. The episode resolved after discontinuing the offending drug.

We performed a literature search on PubMed and Google Scholar databases for IC related to oral phenylephrine use. Only 1 case report linking acute IC to oral phenylephrine use was found.^5^ Table 1 shows the characteristics of our patient compared with the previously reported case by Ward et al (similar word count).

**Table 1.**
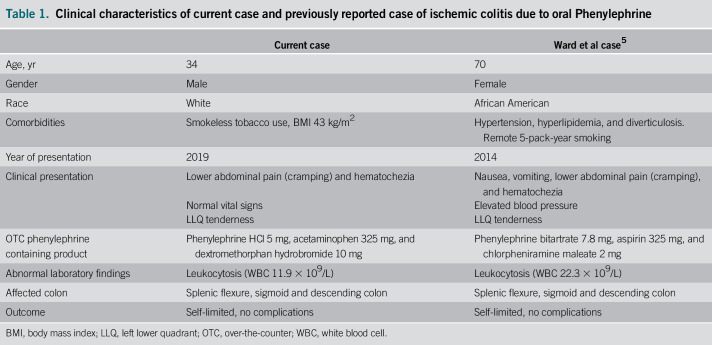
Clinical characteristics of current case and previously reported case of ischemic colitis due to oral Phenylephrine

	Current case	Ward et al case^5^
Age, yr	34	70
Gender	Male	Female
Race	White	African American
Comorbidities	Smokeless tobacco use, BMI 43 kg/m^2^	Hypertension, hyperlipidemia, and diverticulosis. Remote 5-pack-year smoking
Year of presentation	2019	2014
Clinical presentation	Lower abdominal pain (cramping) and hematochezia	Nausea, vomiting, lower abdominal pain (cramping), and hematochezia
	Normal vital signs	Elevated blood pressure
	LLQ tenderness	LLQ tenderness
OTC phenylephrine containing product	Phenylephrine HCl 5 mg, acetaminophen 325 mg, and dextromethorphan hydrobromide 10 mg	Phenylephrine bitartrate 7.8 mg, aspirin 325 mg, and chlorpheniramine maleate 2 mg
Abnormal laboratory findings	Leukocytosis (WBC 11.9 × 10^9^/L)	Leukocytosis (WBC 22.3 × 10^9^/L)
Affected colon	Splenic flexure, sigmoid and descending colon	Splenic flexure, sigmoid and descending colon
Outcome	Self-limited, no complications	Self-limited, no complications

BMI, body mass index; LLQ, left lower quadrant; OTC, over-the-counter; WBC, white blood cell.

Pseudoephedrine—in contrast to phenylephrine—was reported to cause IC in 11 case reports.^6–13^ Pseudoephedrine is a more potent decongestant compared with oral phenylephrine, largely because of its higher systemic bioavailability.^14^ Pseudoephedrine was more widely used before restrictions were placed on its sale to limit its illicit conversion to methamphetamines.^15^ As a result, phenylephrine has largely replaced pseudoephedrine in many OTC cold and allergy medicines.

We conclude that our patient is the first reported case of acute IC due to oral phenylephrine use in a young person. The occurrence of IC with the less potent systemic decongestant phenylephrine in a patient with limited risk factors, and the fact that henylephrine became the more prevalent decongestant, suggests the call for extra caution with OTC decongestants use. Onset of abdominal pain and diarrhea should alarm individuals to promptly discontinue the drug and seek medical attention. Finally, a detailed medication history including the use of OTC products is crucial and can significantly contribute to the patient's management.

## DISCLOSURES

Author contributions: E. El-Alali and T. Alhmoud wrote the manuscript and reviewed the literature. E. El-Alali is the article guarantor.

Financial disclosure: None to report.

Informed consent was obtained for this case report.
